# Investigation into the promoter DNA methylation of three genes (CAMK1D, CRY2 and CALM2) in the peripheral blood of patients with type 2 diabetes

**DOI:** 10.3892/etm.2014.1766

**Published:** 2014-06-06

**Authors:** JIA CHENG, LINLIN TANG, QINGXIAO HONG, HUADAN YE, XUTING XU, LEITING XU, SHIZHONG BU, QINWEN WANG, DONGJUN DAI, DANJIE JIANG, SHIWEI DUAN

**Affiliations:** 1 Zhejiang Provincial Key Laboratory of Pathophysiology, School of Medicine, Ningbo University, Ningbo, Zhejiang 315211, P.R. China; 2 Department of Clinical Medicine, Ningbo Kangning Hospital, Ningbo, Zhejiang 315201, P.R. China

**Keywords:** type 2 diabetes, calcium/calmodulin-dependent protein kinase 1D, cryptochrome 2, calmodulin 2, DNA methylation, promoter

## Abstract

Promoter DNA methylation may reflect the interaction between genetic backgrounds and environmental factors in the development of metabolic disorders, including type 2 diabetes (T2D). Calcium/calmodulin-dependent protein kinase 1D (CAMK1D), cryptochrome 2 (CRY2) and calmodulin 2 (CALM2) genes have been identified to be associated with a risk of T2D. Therefore, the aim of the present study was to investigate the contribution of promoter DNA methylation of these genes to the risk of T2D. Using bisulfite pyrosequencing technology, the DNA methylation levels of the CpG dinucleotides within the CAMK1D, CRY2 and CALM2 gene promoters were measured in 48 patients with T2D and 48 age- and gender-matched healthy controls. The results demonstrated that the promoters of these three genes were hypomethylated in the peripheral blood of all the subjects, and DNA methylation of these three genes did not contribute to the risk of T2D.

## Introduction

Type 2 diabetes (T2D) is a major public health problem in numerous countries (1,2). Genetic and environmental factors are hypothesized to contribute to the initiation and development of the disease (3). Genetic association studies based on genome-wide association and meta-analyses have revealed a number of susceptible genetic polymorphisms associated with the pathogenesis of T2D (2,4–7). However, a number of these polymorphisms are not functional variants and are most likely cotransmitted with the causative polymorphisms in large linkage disequilibrium blocks, and do not directly control the expression of T2D susceptibility genes (8). Evaluating the impact of these genetic-environmental interactions and predicting the personal onset risk of developing T2D remains a challenge.

Epigenetic regulation is sensitive to a number of environmental factors and may provide a bridge between the environment and genetic background (1). As a crucial mechanism in epigenetic regulation, DNA methylation regulates gene expression by transferring a methyl group to cytosine nucleotides via DNA methyltransferase (1). Unlike genetic polymorphisms, DNA methylation levels may alter during numerous processes, including development, tissue differentiation and aging (1). In a monozygotic twins study, the older twins showed more distinct changes according to DNA methylation profiles in the epigenome (9,10). Environmental factors, including nutrition and lifestyle, may influence DNA methylation in mammals. For example, the promoter methylation of certain genes was increased in human primary muscle cells due to the exposure of the free fatty acids palmitate (10). A series of genes in primary metabolic processes were reported to show a differential methylation status in skeletal muscle in patients with T2D compared with normal glucose-tolerant individuals (11). Furthermore, dysregulation of promoter methylation has been found in pancreatic islets from patients with T2D (1). Elucidating the association between promoter methylation and the pathogenesis of T2D is a promising field in the research of metabolic disorders (1,10).

The calcium/calmodulin-dependent protein kinase 1D (CAMK1D) gene encodes a member of the Ca^2+^/calmodulin-dependent protein kinase 1 subfamily of serine/threonine kinases that plays an important role in the regulation of granulocyte function via the chemokine signaling transduction pathway (12,13). Single nucleotide polymorphisms (SNPs) in CAMK1D have been found to be associated with the susceptibility of developing T2D in an east Asian population (14). However, no significant association between the CAMK1D SNP and T2D was found in a study based on European populations (15). Thus, the role of the CAMK1D gene in the pathophysiology of T2D remains unclear (13). The calmodulin 2 (CALM2) gene encodes a member of the human calmodulin family, and polymorphisms in this gene have been associated with dialysis survival in T2D-associated renal disease (16). The cryptochrome 2 (CRY2) gene is a circadian signal gene, whose genetic variation has been associated with T2D and metabolic characteristics (17,18). A recent study investigating monozygotic twins found that the methylation status of CRY2 in subcutaneous adipose tissue differed between the twin with T2D and the healthy twin (18). Further investigations into the association between promoter methylation of the CRY2 gene and T2D in the peripheral blood should be conducted.

Aberrant gene methylation has been identified not only in diseased tissues, but also in human peripheral tissue, including blood lymph cells (8). Detecting the promoter methylation levels of T2D-associated genes in the peripheral blood may be beneficial for the identification of novel diabetic biomarkers with preventative and/or diagnostic values. In the present study, the methylation levels of three candidate genes (CAMK1D, CRY2 and CALM2) in patients with T2D and non-diabetic individuals were investigated to determine the value of this epigenetic marker, with the aim of providing further understanding into the disease etiology of T2D.

## Materials and methods

### Subjects

A total of 48 patients with T2D and 48 age- and gender-matched healthy controls were recruited from the Affiliated Hospital of Ningbo University (Ningbo, China). The characteristics of the individuals are shown in Table I. All the individuals were of Han Chinese origin and had lived in the Ningbo area for at least three generations. Patients with T2D were recruited if their plasma glucose levels were >7.0 mmol/l at fasting or >11.1 mmol/l at 2 h following glucose load (World Health Organization) (19). Healthy individuals were recruited according to the standard of fasting blood glucose of <6.1 mmol/l. None of the controls had a family history of T2D in first-degree relatives or had received any medication. Subjects were excluded from the study if they had hypertension, coronary heart disease, renal inadequacy, drug abuse or any other serious diseases. The study was approved by the Ethics Committee of Ningbo University and written informed consent was obtained from all the subjects. Blood samples were collected in 3.2% citrate sodium-treated tubes and stored at −80°C for DNA extraction.

### Phenotype collection

Blood samples were collected from the antecubital vein into vacutainer tubes containing ethylene diamine tetraacetic acid following fasting for 12 h overnight. Plasma levels of cholesterol, triglyceride, alanine aminotransferase (ALT), uric acid and glucose concentrations were enzymatically measured using the CX7 Analyzer (Beckman Coulter, Inc., Fullerton, CA, USA).

### DNA methylation assay

**Figure 1:**
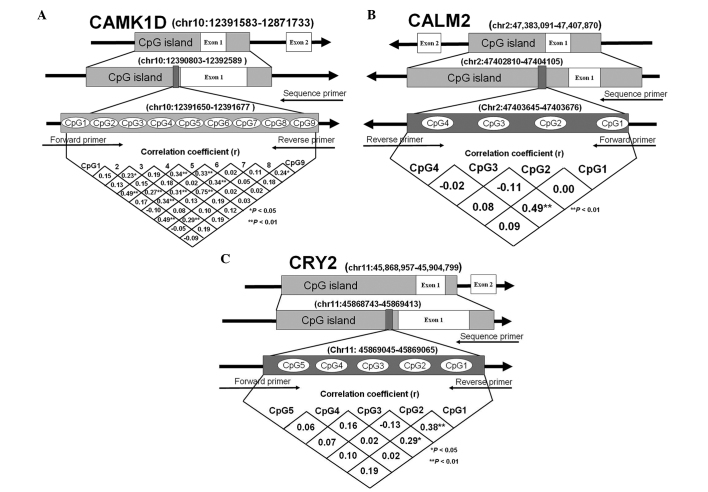
CpG islands within the CAMK1D, CRY2 and CALM2 promoters. The diagrams indicate the correlation coefficients, and (A) nine CpG dinucleotides within CAMK1D, (B) four CpG dinucleotides within CALM2 and (C) five CpG dinucleotides within CRY2. ^*^P<0.05, ^**^P<0.01. P-values were calculated by Pearson test which analyzed the correlation of the methylation levels among the tested CpGs. CALM2, calmodulin 2; CRY2, cryptochrome 2; CAMK1D, calcium/calmodulin-dependent protein kinase 1D.

Human genomic DNA was isolated from peripheral blood samples using a nucleic acid extraction automatic analyzer (Lab-Aid 820; Xiamen, Fujian, China). DNA was quantified using the PicoGreen double strand DNA Quantification kit (Molecular Probes, Inc., Eugene, OR, USA). Bisulfite pyrosequencing technology was used to determine the CpG dinucleotide methylation levels of fragments within the promoters of the CAMK1D, CRY2 and CALM2 genes (Fig. 1). The pyrosequencing assays involved sodium bisulfite DNA conversion chemistry, polymerase chain reaction (PCR) amplification and sequencing by synthesis assay of the target sequence. Sodium bisulfite preferentially deaminates unmethylated cytosine residues to thymines (following PCR amplification), while methyl-cytosines remain unmodified. PCR primers were selected using PyroMark Assay Design software v2.0.1.15 and the amplification primers for the CAMK1D, CRY2 and CALM2 gene promoters are shown in Table II.

### Statistical analysis

Pearson’s χ^2^ test was used to compare the categorical variables, while mean group differences for continuous variables were compared using the Student’s t-test. Pearson’s correlation analysis was applied to determine the associations between the methylation status and metabolic features of the subjects. P<0.05 was considered to indicate a statistically significant difference. All statistical analyses were performed using PASW Statistics 18.0 software (SPSS, Inc., Chicago, IL, USA).

## Results

### Low methylation levels within the CAMK1D, CRY2 and CALM2 promoters

**Figure 2:**
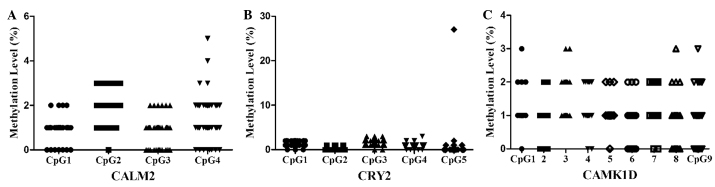
DNA methylation of all the CpGs within the CAMK1D, CRY2 and CALM2 promoters. Methylation levels of each dinucleotide of the (A) four CpGs within CALM2, (B) five CpGs within CRY2 and (C) nine CpGs within CAMK1D. CALM2, calmodulin 2; CRY2, Cryptochrome 2; CAMK1D, calcium/calmodulin-dependent protein kinase 1D.

A total of 48 patients with T2D and 48 age- and gender-matched controls were recruited for the association study, and the methylation levels of three genes (CAMK1D, CRY2 and CALM2) were investigated. In total, four CpG dinucleotides within the CALM2 gene promoter, five CpG dinucleotides within the CRY2 gene promoter and nine CpG dinucleotides within the CAMK1D gene promoter were identified. The correlations between the DNA methylation levels among all the CpGs are shown in Fig. 1 and the characteristics of the subjects are shown in Table I. As shown in Table I, the promoters of the three genes in the peripheral blood exhibited low methylation levels for all the subjects. The DNA methylation information of all the CpGs within the CAMK1D, CRY2 and CALM2 promoters is shown in Fig. 2.

### Statistical analysis of clinical phenotypes

No statistically significant differences in clinical phenotypes, including age, body mass index, total cholesterol and uric acid, were observed between the T2D and controls patients (Table I; P>0.05). However, the glucose level in patients with T2D was significantly higher compared with the control patients (Table I; P<0.001), and a similar result was observed in the breakdown analysis by gender. The levels of ALT and total triglycerides were higher in patients with T2D (Table I; P=0.028 and P=0.034, respectively). However, in the breakdown analysis by gender, no differential level of total triglycerides was observed. Notably, higher levels of ALT in female patients with T2D were observed (Table III; P=0.019).

## Discussion

Epigenetic regulation is involved in numerous types of human diseases, and epigenetic biomarkers have been reported to be associated with pathological processes, of which a number have potential value as laboratory diagnostic tools (20–23). DNA methylation, as one of the most studied epigenetic regulatory mechanisms, is essential in the interaction between genetic and environmental factors (10,20). DNA methylation usually occurs in the CpG islands of functional gene promoter regions (20), and this type of epigenetic modification was originally considered to be stable *in vitro* and function as a tissue-specific feature in disease (10,24,25). A number of disease-associated DNA methylation variations have been identified in a diverse range of tissues, including the peripheral blood (8,10,20,26).

T2D is a major metabolic disease, which physiological and pathological states may be regulated by epigenetic modifications that control gene expression (10). Hundreds of differential DNA methylation CpGs of genes promoters have been identified in genomic DNA isolated from pancreatic islets using Infinium methylation assay technology (1). These disease-specific methylation signatures also exist in human accessible peripheral tissues (8). A prospective study found that significant hypomethylation of young individuals was associated with a higher risk of developing T2D in later life (8). In the present study, the DNA promoter methylation levels of three T2D candidate genes (CALM2, CRY2 and CAMK1D) in the peripheral blood were investigated, providing further information regarding DNA promoter methylation regulation in T2D.

Low levels of DNA promoter methylation were identified in the three genes in the healthy subjects and patients with T2D (Table I). The majority of the methylation correlation coefficients of the CpGs within these genes were also not significant (Fig. 1). Two reasons may explain these observations. In epigenetics, not all candidate genes are directly regulated by DNA methylation, with other epigenetic factors, including non-coding RNA and histone variants, also involved in the regulation of gene expression (20,23). Since T2D is a polygenic disease, the low DNA promoter methylation levels of CALM2, CRY2 and CAMK1D genes may indicate that DNA promoter methylation has no direct effect on gene function. In addition, the methylation status was only investigated in the peripheral blood, and DNA promoter methylation modification has been demonstrated to exhibit a tissue-specific methylation pattern (25). The insulin-2 gene, an additional candidate gene for diabetes, was found to be unmethylated in β cells, but methylated in other tissues (25). The identification of DNA methylation profiling in pancreatic islets revealed that differences in the methylation status of certain candidate genes between T2D and non-diabetic patients were not present in blood cells (1). The two aforementioned reasons may explain the low DNA methylation levels of CALM2, CRY2 and CAMK1D observed in the present study.

In conclusion, low methylation levels of CALM2, CRY2 and CAMK1D were observed in the peripheral blood of the healthy controls and T2D patients. These observations indicate that DNA methylation may not be the primary regulatory mechanism of CALM2, CRY2 and CAMK1D in T2D, and these methylation loci may not be regarded as biomarkers for T2D. The present study provides further information with regard to the epigenetic mechanisms and may provide reference value in genetic-based pharmacological development for future T2D treatments.

## Figures and Tables

**Table I:** Characteristics of the subjects (n=96).

Characteristics	Value	Range	T2D	Controls	P-value
Age (years)	59.2±7.5	35–69	59.2±7.5	59.2±7.5	1.000
Gender (M/F)	48/48	-	-	-	-
BMI (kg/m^2^)	23.71±3.28	17.15–42.96	24.17±4.18	23.18±1.64	0.146
Total cholesterol (mmol/l)	5.19±0.96	2.95–7.90	5.34±0.83	5.05±1.06	0.140
Total triglycerides (mmol/l)	1.60±1.36	0.40–9.92	1.90±1.69	1.31±0.82	0.034
Glucose (mmol/l)	6.76±2.65	4.38–22.84	8.31±2.91	5.22±0.92	0.000
ALT (IU/l)	21.5±15.9	5.0–99.0	25.1±18.5	18.0±12.1	0.028
Uric acid (μmol/l)	294.9±81.1	132.0–531.0	289.3±70.5	300.6±90.9	0.499
CALM2 methylation (%)					
CpG1	0.96±0.38	0–2	1.02±0.40	0.88±0.33	0.099
CpG2	2.01±0.50	0–3	2.18±0.39	1.79±0.55	0.001
CpG3	0.92±0.48	0–2	1.00±0.53	0.82±0.39	0.087
CpG4	1.40±0.86	0–5	1.11±0.58	1.79±1.02	0.001
CRY2 methylation (%)					
CpG1	1.25±0.53	0–2	1.13±0.41	1.44±0.65	0.022
CpG2	0.80±0.41	0–1	0.79±0.41	0.80±0.41	0.961
CpG3	1.61±0.63	0–3	1.64±0.63	1.56±0.65	0.621
CpG4	0.98±0.42	0–3	1.05±0.39	0.88±0.44	0.110
CpG5	1.14±3.32	0–27	0.64±0.54	1.92±5.24	0.134
CAMK1D methylation (%)					
CpG1	1.04±0.29	0–3	1.04±0.20	1.04±0.36	1.000
CpG2	0.75±0.48	0–2	0.90±0.31	0.60±0.57	0.003
CpG3	1.53±0.54	1–3	1.75±0.48	1.31±0.51	0.000
CpG4	1.04±0.29	0–2	1.10±0.31	0.98±0.25	0.032
CpG5	1.02±0.20	0–2	1.04±0.20	1.00±0.21	0.320
CpG6	0.96±0.32	0–2	1.02±0.14	0.90±0.43	0.057
CpG7	1.04±0.29	0–2	1.06±0.38	1.02±0.14	0.480
CpG8	0.92±0.45	0–3	1.02±0.25	0.81±0.57	0.023
CpG9	0.82±0.54	0–3	1.00±0.41	0.65±0.60	0.001

Results are expressed as the mean ± standard error.

a n=86 (46 patients with T2D vs. 40 control patients).

T2D, type 2 diabetes; BMI, body mass index; ALT, alanine aminotransferase; CALM2, calmodulin 2; CRY2, cryptochrome 2; CAMK1D, calcium/calmodulin-dependent protein kinase 1D; M, male; F, female.

**Table II:** Primers for the promoter CpG methylation analysis.

Gene	Sequence
CALM2	
Forward primer	5′-AGGAGGAGTTGTTGGAGAATATGA-3′
Reverse primer	5′-biotin- ACTACCCCCCTAACCCCTCT-3′
Sequencing primer	5′-GTTTTGAGTGTTTAGGTAAGG-3′
CRY2	
Forward primer	5′-GGGGTGGTTGGAGTAGTTTGG-3′
Reverse primer	5′-biotin-AATCCCCTCACCTCCATC-3′
Sequencing primer	5′-GGAGTAGTTTGGATAGTTA-3′
CAMK1D	
Forward primer	5′-GGAGGTAAGAAAGTAGTAGAAAGTGA-3′
Reverse primer	5′-biotin-CCTCCTCTACAATTTCCTCTT-3′
Sequencing primer	5′-GAGTTAGGGAGGGAT-3′

CALM2, calmodulin 2; CRY2, cryptochrome 2; CAMK1D, calcium/calmodulin-dependent protein kinase 1D.

**Table III:** Characteristics of the subjects by gender.

Characteristics	T2D	Controls	P-value
Males (n=48)			
Age (years)	59.1±8.7	59.1±8.7	
BMI (kg/m^2^)^a^	24.92±5.17	23.10±1.21	0.124
Total cholesterol (mmol/l)	5.06±0.74	4.86±1.11	0.464
Total triglycerides (mmol/l)	1.81±1.56	1.38±0.90	0.254
Glucose (mmol/l)	8.59±3.49	4.94±0.34	0.000
ALT (IU/l)	30.4±23.8	21.1±15.9	0.119
Uric acid (μmol/l)	304.7±70.6	346.5±82.7	0.066
Females (n=48)			
Age (years)	59.4±6.4	59.4±6.4	
BMI (kg/m^2^)^b^	23.49±2.97	23.25±1.93	0.747
Total cholesterol (mmol/l)	5.62±0.83	5.24±1.00	0.161
Total triglycerides (mmol/l)	1.98±1.85	1.23±0.75	0.071
Glucose (mmol/l)	8.04±2.22	5.49±1.20	0.000
ALT (IU/l)	19.8±8.6	14.8±4.8	0.019
Uric acid (μmol/l)	273.9±68.3	254.6±75.0	0.357

a n=39 (22 patients with T2D vs. 17 control patients);

b n=47 (24 patients with T2D vs. 23 control patients).

T2D, type 2 diabetes; BMI, body mass index; ALT, alanine aminotransferase; SE, standard error.

## Abbreviations

T2D: type 2 diabetes
CAMK1D: calcium/calmodulin-dependent protein kinase 1D
CRY2: cryptochrome 2
CALM2: calmodulin 2
CI: confidence interval
ALT: alanine aminotransferase
SNP: single nucleotide polymorphism
PCR: polymerase chain reaction
